# Effects of N-acetyl-cysteine supplementation on sperm quality, chromatin integrity and level of oxidative stress in infertile men

**DOI:** 10.1186/s12958-019-0468-9

**Published:** 2019-02-16

**Authors:** Rahil Jannatifar, Kazem Parivar, Nasim Hayati Roodbari, Mohammad Hossein Nasr-Esfahani

**Affiliations:** 1 Department of Biology, Science and Research Branch, Islamic Azad University, Tehran, Iran; 2 Department of Reproductive Biotechnology, Reproductive Biomedicine Research Center, ACECR, Royan Institute for Biotechnology, Isfahan, Iran

**Keywords:** Infertility, N-acetyl-cysteine, Oxidative stress, Asthenoteratozoospermia

## Abstract

**Background:**

Infertile men have higher levels of semen reactive oxygen species (ROS) than fertile men. High levels of semen ROS can cause sperm dysfunction, sperm DNA damage and reduced male reproductive potential. This study investigated the effects of supplementation with N-acetyl-cysteine (NAC) on the sperm quality, chromatin integrity and levels of oxidative stress in infertile men.

**Methods:**

The study was carried out in the unit of ACECR Infertility Research Center, Qom, Iran. The patients consisted of 50 infertile men with asthenoteratozoospermia who received NAC (600 mg/d) orally for 3 months, after which they were compared with pre-treatment status. Semen was analyzed according to WHO (2010), followed by the assessment of protamine content [chromomycin A3 (CMA3)] and DNA integrity [terminal deoxynucleotidyl transferase-mediated dUTP nick-end labeling (TUNEL)]. Oxidative stress markers, i.e. total antioxidant capacity (TAC) and malondialdehyde (MDA), as well as hormonal profile (LH, FSH, Testosterone and Prolactin) were determined by ELISA kit.

**Results:**

After NAC treatment, patients’ sperm count and motility increased significantly whereas abnormal morphology, DNA fragmentation and protamine deficiency showed significant decreases compared to pre-treatment levels (*P* < 0.05). Hormonal profile improvement was associated with lowered FSH and LH levels and increased amount of testosterone (*P* < 0.05). TAC significantly increased and MDA decreased with an inverse significant correlation between TAC and MDA (*P* < 0.05).

**Conclusion:**

NAC oral supplementation may improve sperm parameters and oxidative/antioxidant status in infertile males.

## Background

Oxidative stress and reactive oxygen species (ROS), known as free radicals, are oxidizing agents with a high reactive capacity. ROS may have either endogenous or exogenous origin and may cause defective spermatogenesis and male infertility [1]. Many environmental, physiological, and genetic factors have been implicated with sperm functions and infertility [2]. In particular, oxidative stress (OS) has been suggested to affect male fertility and the physiology of spermatozoa [3]. In physiological conditions, spermatozoa produce little ROS, which are required for sperm physiology (sperm hyperactivation, capacitation, acrosome reaction) and also for natural fertilization [4].

Increased pathological ROS generation leads to sperm dysfunction (lipid peroxidation), decreased semen quality and sperm DNA damage [5]. In fact, oxidative stress damages sperm genes with the occurrence of single- and/or double stranded DNA [6]. Therefore, scavenging excess ROS is essential for normal spermatogenesis and fertilization [7].

In infertile men’s semen, leukocytes and immature or abnormal spermatozoa are often the two main sources of ROS [8]. Spermatozoa are susceptible to oxidative damage because their plasma membranes are rich in polyunsaturated fatty acids and have low concentrations of scavenging enzymes [9]. At the same time, antioxidants, which protect the cell from excessive ROS-induced lipid peroxidation, are also present in the ejaculate [10]. Antioxidant capacity in the idiopathic infertile male population is lower than that of fertile men, who exhibit significantly greater seminal ROS production [11]. However, it is unclear whether reduced semen antioxidant capacity necessarily causes sperm dysfunction (including sperm DNA damage) [12, 13]. The association between sperm DNA damage and semen ROS is the basis for the use of antioxidants in the treatment of sperm DNA damage and sperm quality. Dietary antioxidants may also have positive effects on sperm parameters [14].

N-acetyl-cysteine (NAC), a derivative of amino acid L-cysteine, is currently used mainly as an antioxidant [15]. NAC also contributes to glutathione (GSH) synthesis [16] and may help restore the depleted pool of GSH often caused by oxidative stress and inflammation [17, 18]. NAC has free radical scavenging activity both in vivo [19] and in vitro [20, 21]. In addition, daily treatment with NAC results in a significant improvement in sperm motility in comparison to placebo [22]. Comhaire et al. also found that NAC improved sperm concentration and acrosome reaction while reducing ROS and oxidation of sperm DNA [23]. Based on the above, the present study was conducted to investigate the effects of daily oral NAC supplementation on the quality of semen parameters, chromatin integrity and reproductive hormones in asthenoteratozoospermic men.

## Methods

### Study design

This study was conducted as a randomized, blinded clinical trial. A total of 50 infertile men with terato-asthenozoospermia and a mean age of 25–40 years were enrolled in the unit of ACECR Infertility Research Center, Qom, Iran, in 2018. This prospective clinical trial was approved by the Ethics Committee for Research Involving Human Subjects at Azad Medical University and was registered in the Iranian website (www.irct.ir) for clinical trials registration (http://www.irct.ir: IRCT20170830035998N4). All individuals gave informed consent prior to participation in the study.

Inclusion criteria included the infertile couples with no previous report of pregnancy, normal female partner and male partner defined as having Asthenoteratozoospermia based on World Health Organization (WHO, 2010) criteria [24]. Infertile patients with well-known pathologic features such as varicocele, leukospermia, hormonal abnormalities, and/or obstruction, the presence of cryptorchidism, vasectomy, abnormal liver function, cigarette smoking, alcohol consumption, anatomical disorders, Klinefelter’s syndrome, cancer, fever in the 90 days prior to sperm analysis, seminal sperm antibodies, were excluded from the study.

### Intervention and assessment

Male age and duration of infertility were recorded at time of the study. In addition, male height (m), weight (kg) and body mass index (BMI, kg/m^3^) were recorded and compared before and after intervention. The subjects consumed NAC 600 mg/day by oral route for 3 months [19, 22]. Variables including seminal parameters, DNA fragmentation index, chromatin maturity, total antioxidant capacity, lipid peroxidation and hormonal parameters (LH, FSH, Testosterone and Prolactin) were measured before and after the intervention.

### Semen analysis

Semen samples were obtained through masturbation after 3–5 days of sexual abstinence and allowed to liquefy at room temperature. Semen parameters (volume, sperm count, progressive and non-progressive motility and normal morphology) were evaluated according to WHO guidelines (2010). Briefly, sperm motility was assessed by the Computer Aided Sperm Analysis (CASA) system (LABOMED, SDC313B, Germany), which defined sperm as progressive, non-progressive and immotile. Normal morphology was assessed by Papanicolaou staining [25] and subjects with less than 4% normal sperm morphology were considered as teratozospermic according to WHO criteria. Sperm count was evaluated by a sperm counting chamber and expressed as million/ml.

### Hormonal analysis

The peripheral blood sample was taken from each patient and was immediately centrifuged for 10 min at 3000 rpm (Hettich, EBA20, UK) and serum samples stored at − 70 °C for future evaluation and analysis. The serum levels of follicle stimulating hormone (FSH;mIU/ml, Cat.N.DE1288), luteinizing hormone (LH; mIU/ml, Cat.N.DE1289), prolactin (PRL; ng/ml, Cat.N.DE1291) and total testosterone (TT; ng/ml, Cat.N.DE1559) in all samples were measured using ELISA enzyme immunoassay (Demeditec Diagnostics GmbH, Germany) for hormonal profile.

### Assessment of lipid peroxidation and seminal total antioxidant capacity

Seminal Malondialdehyde (MAD) was assumed as a direct measure of lipid peroxidation and detected by Abnova ELISA Kit (Cat.N.KA3736, Abnova Corporation, Taiwan) at a detection range of 0.125–2 mM (125–2000 mmol/L). Seminal total antioxidant capacity (TAC) was measured using a commercially available kit (Zell Bio GmbH, Wurttemberg, Germany).

### Assessment of DNA fragmentation

Sperm DNA fragmentation was evaluated by terminal deoxynucleotidyl transferase dUTP nick-end labeling (TUNEL) assay using the in situ cell death detection kit (Roche, Mannheim, Germany) [26]. Five hundred sperms per slide were assessed under a fluorescence microscope (BX51, Olympus, Japan) at 100x magnification. Sperm with red heads were considered to have intact DNA, and those with green heads were assumed to have fragmented DNA.

### Assessment of protamine deficient sperms

Protamine deficiency was determined with CMA3 staining according to Nasr-Esfahani et al. [27]. Briefly, PBS-washed spermatozoa were fixed with Carnoy’s solution (3:1, methanol and acetic acid) at 4 °C for 5 min followed by preparation of smears. Afterwards, each slide was treated with 100 μL of 0.25 mg ml^− 1^ CMA3 (Sigma Co.) in a dark and humid condition for 20 min. The slides were then washed with phosphate buffer solution (PBS) and kept at 4 °C overnight until examination. Five hundred sperm per slide were evaluated under a fluorescence microscope (BX51, Olympus, Japan). Spermatozoa with bright yellow staining were considered as protamine deficient (CMA3 positive), and those with dull yellow staining were considered to have normal amounts of protamines (CMA3 negative).

### Statistical analysis

All data were presented as mean ± standard error of mean (SEM). Normal distribution of data was assessed by Kolmogorov-Smirnov Z test. Data before and after NAC treatment were compared by the paired t-test via the statistical package for social studies (SPSS software, Version 20 Chicago, IL, USA). Mean differences were considered statistically significant at *P* < 0.05.

## Results

### Clinical and demographic characteristics

The mean age of participants was 34.7 ± 4.17 years. The duration of infertility was 2.1 ± 0.2 years. Male mean values for weight, height and BMI did not significantly change after the intervention (Table 1).

**Table 1:** Comparison of age, duration of infertility, weight, height, and BMI before and after treatment with NAC. No differences were observed after treatment with NAC

Variable	Before NAC (*n* = 50)	After NAC (*n* = 50)	*P*-Value
Duration of infertility (Y)	2.1 ± 0.2	2.1 ± 0.2	0.329
Weight (kg)	85.96 ± 1.12	85.72 ± 1.11	0.141
Height (cm)	164.53 ± 5.33	164.53 ± 5.33	0.111
BMI (kg/m^2^)	29.19 ± 0. 23	29.13 ± 0.23	0.129

Data are shown as mean ± SD. Analysis was performed by the Paired t-test; NAC: N-acetyl-L-cysteine; BMI; Body Mass Index

### Comparison of seminal parameters before and after NAC treatment

Sperm parameters are presented in Table 2. NAC supplementation significantly improved sperm total motility (31.42% ± 0.60 vs. 35.18% ± 1.21; *P* = 0.01), progressive motility (20.60% ± 0.77 vs. 24.54% ± 1.08; *P* = 0.001) and sperm concentration (46.52 10^6^/mL ±1.80 vs. 51.06 10^6^/mL ± 2.51; *P* = 0.02). Mean percentage of non-progressive motility (19.10% ± 0.76 vs. 16.00% ± 1.75; *P* = 0.01), immotile sperm (63.00% ± 0.71 vs. 61.82% ± 0.79; P = 0.01) and abnormal morphology (98.12% ± 0.11 vs. 94.02% ± 0.16; *P* = 0.001) showed a significant improvement after treatment.

**Table 2:** Comparison of semen parameters before and after treatment with NAC. Sperm parameters significantly improved after treatment with NAC compared to before treatment

Sperm Parameters	Before NAC (*n* = 50)	After NAC (*n* = 50)	P- Value
Volume (ml)	3.58 ± 0.12	4.02 ± 0.18^*^	***P*** **= 0.01**
Total Motility (%)	31.42 ± 0.60	35.18 ± 1.21^*^	***P*** **= 0.01**
Progressive motility (a + b) (%)	20.60 ± 0.77	24.54 ± 1.08^*^	***P*** **= 0.001**
Non-Progressive motility (%)	19.10 ± 0.76	16.00 ± 1.19^*^	***P*** **= 0.01**
Immotile Sperm (%)	63.00 ± 0.71	61.82 ± 0.79^*^	***P*** **= 0.01**
Sperm concentration (10^6^/mL)	46.52 ± 1.80	51.06 ± 2.51^*^	***P*** **= 0.02**
Abnormal Morphology (%)	98.12 ± 0.11	96.02 ± 0. 16^*^	***P*** **= 0.001**

NAC: N-acetyl-cysteine. Significant differences in bold

### Comparison of DNA fragmentation and protamine deficiency before and after NAC treatment

**Fig. 1:**
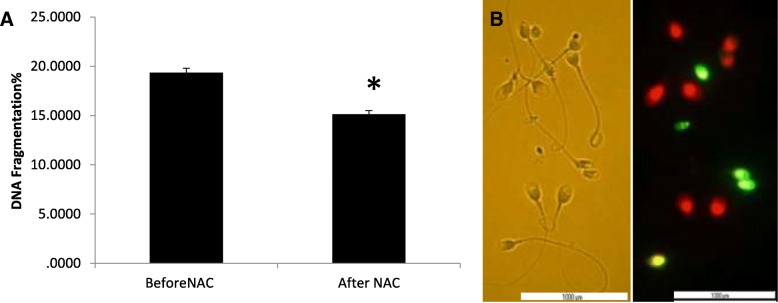
**a** Comparison of sperm DNA fragmentation before and after treatment with NAC (N = 50). Percentage of DNA fragmentation in sperm significantly decreased after treatment with NAC compared to before treatment. **b** Light microscope observation of sperm morphology **c** DNA fragmentation was assessed by TUNEL assay. Red-staining sperm have intact DNA, while green-staining sperm have DNA fragmentation. Statistical analysis was performed by the paired t-test. NAC; N-acetyl-L-cysteine. * *P* < 0.05

**Fig. 2:**
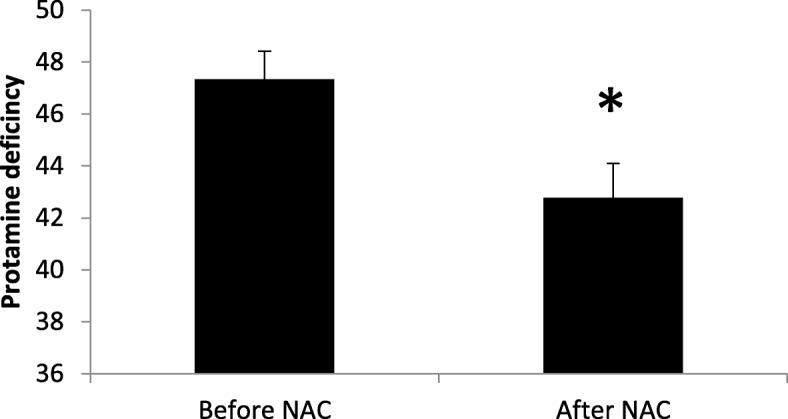
Comparison of sperm protamine deficiency before and after treatment with NAC (*N* = 50). Percentage of protamine deficiency in sperm significantly decreased after treatment with NAC compared to before treatment. Protamine deficiency was assessed by CMA3 staining. Statistical analysis was performed by the paired t-test. NAC: N-acetyl-L-cysteine. * *P* < 0.05

The mean percentage of DNA fragmentation assessed by TUNEL was 19.34% ± 0.47 at baseline and significantly decreased after treatment with NAC (15.14% ± 0.46; P = 0.001), (Fig. 1). Similarly, the mean percentage of CMA3 positive or protamine deficient sperm significantly decreased after treatment (47.33% ± 1.09 vs. 42.77% ± 1.28; *P* = 0.009) (Fig. 2).

### Comparison of hormonal profile before and after NAC treatment

Mean values of FSH (4.14 mU/ml ± 0.17 vs. 3.67 mU/ml ± 0.19, *P* = 0.01) and LH (4.20 mU/ml ± .06 vs. 4.07 mU/ml ± .054, *P* = 0.04) significantly decreased while the mean value of testosterone significantly increased after treatment with NAC (P = 0.01). PRL did not show significant changes (*P* = 0.193) (Table 3).

**Table 3:** Comparison of FSH, LH, Testosterone, and prolactin before and after treatment with NAC. Significant reduction of serum LH, FSH and, significant increase of serum testosterone levels was observed after treatment with NAC compared to before treatment

Hormonal analysis	Before NAC (*n* = 50)	After NAC (*n* = 50)	*P*-Value
FSH (mIU/mL)	4.14 ± 0.17	3.67 ± 0.19	**0.01**
LH (mIU/mL)	4.20 ± .060	4.07 ± .054	**0.04**
Testosterone (ng/mL)	3.87 ± 0.15	4.37 ± 0.17	**0.01**
PRL (ng/ml)	10.61 ± 0.37	10.60 ± 0.37	0.193

FSH: Follicle stimulating hormone, LH: Luteinizing hormone, PRL: Prolactin, NAC: N-acetylcysteine. Significant differences in bold

### Comparison of total antioxidant capacity and level of lipid peroxidation before and after treatment with NAC

**Fig. 3:**
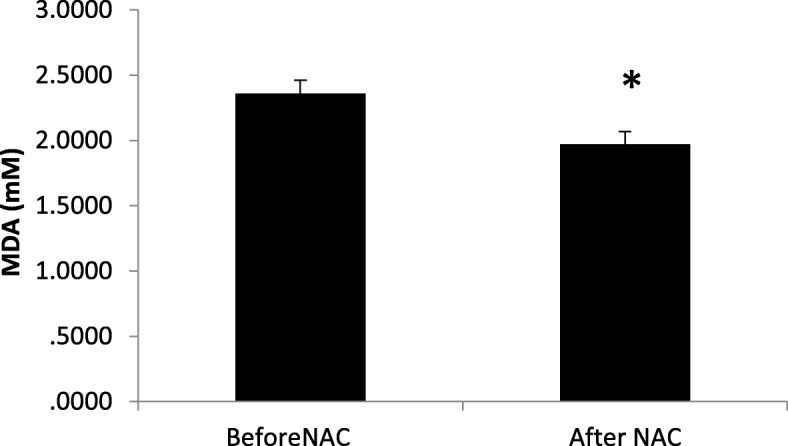
Comparison of seminal malondialdehyde (MDA) before and after treatment with NAC (*N* = 50). Mean of seminal MDA decreased significantly after treatment with NAC compared to before treatment. Statistical analysis was performed by the paired t-test. NAC: N-acetyl-L-cysteine. * *P* < 0.05

**Fig. 4:**
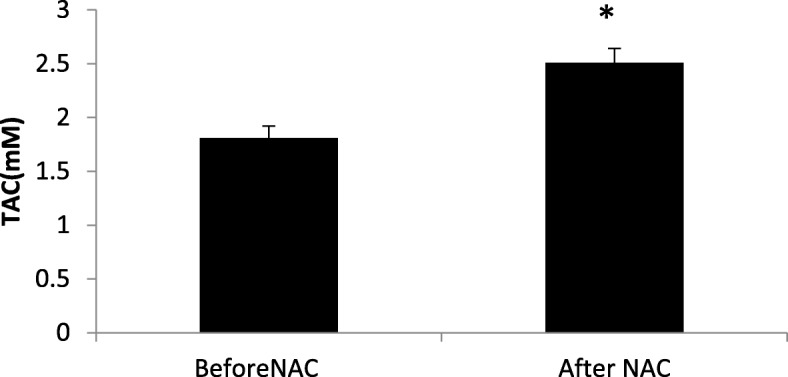
Comparison of Total Antioxidant Capacity (TAC) in seminal plasma before and after treatment with NAC (N = 50). Mean of TAC significantly increased after treatment with NAC compared to before treatment. Statistical analysis was performed by the paired t-test. NAC; N-acetyl-L-cysteine. * *P* < 0.05

MDA levels were significantly lower after NAC treatment (2.36 ± 0.10 vs. 1.97 ± 0.097; P = 0.01 - Fig. 3) and TAC levels were significantly higher (1.82 ± 0.11 vs. 2.51 ± 0.13; *P* = 0.01 - Fig. 4).

### Correlations between study parameters

**Fig. 5:**
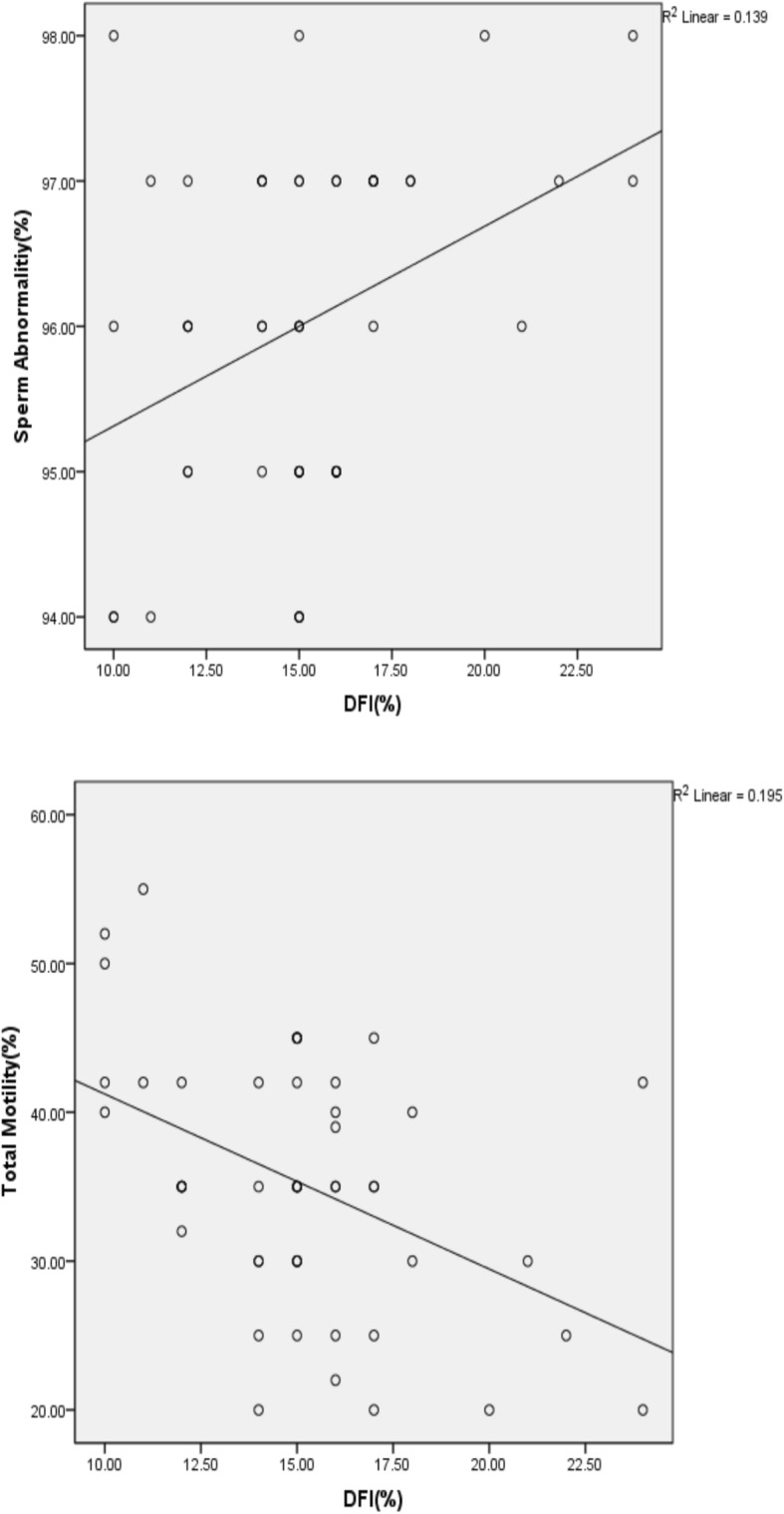
Relationship between sperm DNA fragmentation and sperm motility or abnormality. **a**: Correlation between of DNA fragmentation and sperm abnormality (r = 0.373, P = 0.008). **b**: Correlation between of DNA fragmentation and sperm total motility (r = − 0.442, P = 0.001)

The percentage of DNA fragmentation correlated positively with sperm abnormality (*r* = 0.373, *p* = 0.008) (Fig. 5a) and negatively with sperm total motility (*r* = − 0.442, *p* = 0.001) (Fig. 5b).

After NAC treatment, sperm motility correlated positively with TAC and negatively with MDA. Sperm abnormality, DNA fragmentation and protamine deficiency showed a negative correlation with TAC and a positive correlation with MDA (Table 4).

**Table 4:** Correlation between sperm parameters and chromatin status with total antioxidant capacity (TAC) and malondialdehid (MAD)

Correlations	TAC (mM)		MDA (mM)	
	r	*P*-value	r	*P*-value
Sperm Abnormality (%)				
Before	−0.339	**0.01**	0.301	**0.03**
After	−0.393	**0.005**	0.333	**0.01**
Sperm concentration (10^6^/mL)				
Before	−0.097	0.503	0.045	0.759
After	**−**0.072	0.618	0.106	0.465
Total Motility (%)				
Before	0.384	**0.006**	−0.319	**0.02**
After	0.455	**0.001**	−0.435	**0.002**
CMA3 + (%)				
Before	−0.441	**0.001**	0.327	**0.02**
After	−0.438	**0.001**	0.359	**0.01**
DNA fragmentation (%)				
Before	−0.363	**0.01**	0.361	**0.01**
After	−0.456	**0.001**	0.365	**0.009**

CMA3; Chromomycin A3. TAC: Total Antioxidant Capacity, MDA: Malondialdehyde, NAC: N-acetylcysteine

Significant differences in bold

## Discussion

The present study assessed the effects of NAC supplementation on semen parameters and antioxidant status in asthenoteratozoospermic men partners of infertile couples. The three-month supplementation with NAC significantly improved sperm parameters (count, motility and normal morphology) from pre-treatment baseline. Based on our findings and other studies [19, 22], medical therapy with oral antioxidants can improve the quality of semen parameters. Recently, Buhling et al. [28] reported that male infertility is associated with a decreased intake of some antioxidant nutrients, such as vitamins A, C, and E, carnitine, folate, zinc, and selenium. N-acetylcysteine (NAC) is a thiol-based antioxidant that plays an important role in the protection of cellular constituents against oxidative damage. The hypothetical action of NAC originates from its ability to stimulate and sustain intracellular levels of reduced glutathione and also to detoxify ROS [15]. Safarinejad et al. [22] also reported significant improvements in all semen parameters in subjects receiving selenium or NAC, with an additive effect on the combination group. The effects of NAC on ROS and sperm motility may be mediated by a modulation of mitochondrial activity: 2 mM NAC, alone or combined with methyl donors, B6 and zinc was shown to protect human sperm mitochondria from the decay of their membrane potential and to decrease mitochondrial ROS production in bovine sperm following in vitro incubation up to 180 min [29].

The association between sperm DNA damage and ROS is the basis for the use of antioxidants in the hope of reducing sperm DNA damage. Both exogenous and endogenous ROS can induce sperm DNA damage in vitro, confirming that ROS may play a role in the etiology of sperm DNA damage in infertile men [30, 31] .

There are a few reports on the effects of dietary antioxidant supplementation on sperm DNA integrity. Most of such clinical studies have evaluated men with high levels of sperm DNA damage. Treatment with antioxidant supplements is generally associated with reduced levels of sperm DNA damage and/or improved fertility potential [32, 33]. GSH is a critical intracellular antioxidant that helps limit the impact of oxidative stress and protect vital cellular components (lipids, proteins, DNA) against harmful peroxidation. NAC effects on GSH rely on the presence of the free sulfyhydryl group as a ready source of reducing equivalents to quench radical species [16]. Likewise, we show here that supplementation of infertile individuals with 600 mg/day of NAC significantly reduces DNA fragmentation from 19.34 ± 0.47 before treatment to 15.14 ± 0.46 after treatment and also improves sperm protamine content.

In addition, significant inverse correlations were observed between the percentage of TUNEL-positive cells versus sperm motility or morphology, indicating that NAC protects sperm chromatin integrity and reduces DNA fragmentation. The improved DNA integrity could be partly related to enhanced chromatin maturity, i.e. the improved replacement of histones with protamine. Higher amounts of protamines in sperm mean that a relaxed chromatin state has been converted to a compact and stable structure that reduces the accessibility of ROS to DNA. These observations are in line with previous studies indicating that a correct P1:P2 ratio reflects a proper replacement of histones with protamines, which correlates significantly with the rate of DNA fragmentation [34]. NAC may benefit protamination by feeding the one-carbon metabolism, which, besides increasing GSH and antioxidant capacity of sperm, provides activated methyl groups for epigenetic DNA reprogramming [35].

Spermatogenesis is highly controlled by the hormonal milieu of the testis and any alteration in the hormonal profile, besides influencing the rate and quality of spermatogenesis, may deeply affect chromosomal ploidy and integrity of sperm chromatin [36]. The increased pre-treatment gonadotropin levels (FSH and LH) found in our patients are likely related to a state of hypogonadism, causing aberration in the process of spermatogenesis, which improves with NAC supplementation as confirmed by the increase in serum testosterone levels. These results align with a previous study that reported improved serum testosterone after supplementation with NAC [22]. Increased testosterone provides a negative feedback to the hypothalamus and pituitary, leading to reductions in both GnRH pulse frequency and pituitary responsiveness to GnRH, ultimately resulting in reduced gonadotropin release [37].

Bidmeshkipour et al. [38] reported that TAC levels in the seminal plasma of asthenospermic men were significantly lower than those of healthy men, accordingly, the effect of NAC on MDA and TAC was investigated in the seminal plasma. Our results showed a significant decline in concentrations of MDA, a specific marker of lipid peroxidation, while TAC significantly increased after treatment with NAC. Thus, NAC reduced the severity of oxidative stress leading to reduced lipid peroxidation and DNA fragmentation as verified by MDA and TUNEL assay. The improved DNA maturity assessed by CMA3 staining was an indirect effect of NAC. Indeed, oxidative stress hampers chromatin reprogramming including DNA methylation, which has important consequences on the formation of the protamine shield protecting DNA from oxidative stress and thereby DNA damage.

In addition, TAC correlated positively with markers of sperm quality such as motility and negatively with markers of sperm damage including abnormal morphology, protamine deficiency and DNA fragmentation. As expected, opposite correlations were found for MDA, a marker of lipid peroxidation. Taken together, this correlation analysis emphasizes the role of oxidative stress and anti-oxidant capacity in the quality of sperm benefiting from NAC supplementation [39]. In addition to in vivo supplementation of NAC, this antioxidant has also been used to protect sperm from cryo-injury. Despite, numerous studies suggesting NAC can protect sperm from cro-injury but similar effect was not observed in all species [40].

## Conclusion

ROS appears to play an important role in the generation of sperm DNA damage, impairment of semen parameters, and failure of sperm functions leading to male supplementation with NAC for at least 3 months in asthenoteratozoospermic men partners of infertile couples, who benefited from reverting these damages, likely due to its positive effect on the antioxidant defenses.

## Abbreviations

BMI: Body Mass Index
CMA3: Chromomycin A3
FSH: Follicle stimulating hormone
GnRH: Gonadotropin releasing hormone
LH: Luteinizing hormone
MDA: Malondialdehyde
NAC: N-Acetylcysteine
PRL: Prolactin
ROS: Reactive oxygen species
T: Testosterone
TAC: Total antioxidant capacity
